# Open access: changing global science publishing

**DOI:** 10.3325/cmj.2013.54.403

**Published:** 2013-08

**Authors:** Armen Yuri Gasparyan, Lilit Ayvazyan, George D. Kitas

**Affiliations:** 1 Departments of Rheumatology and Research and Development, Dudley Group NHS Foundation Trust (A Teaching Trust of University of Birmingham), Russells Hall Hospital, Dudley, United Kingdom *a.gasparyan@gmail.com*; 2 Department of Medical Chemistry, Yerevan State Medical University, Yerevan, Armenia; 3 Arthritis Research UK Epidemiology Unit, University of Manchester, Manchester, UK

## Abstract

The article reflects on open access as a strategy of changing the quality of science communication globally. Successful examples of open-access journals are presented to highlight implications of archiving in open digital repositories for the quality and citability of research output. Advantages and downsides of gold, green, and hybrid models of open access operating in diverse scientific environments are described. It is assumed that open access is a global trend which influences the workflow in scholarly journals, changing their quality, credibility, and indexability.

Access to scientific resources is essential for analyzing available evidence-base, designing research studies, and writing scholarly papers. It is equally important for reviewing and editing journal submissions and books, which require validation of facts, references, and tracking of redundant or plagiarized material. For academics, broad access to periodicals, theses, books, and presentations is an indispensable tool for upgrading knowledge base and translating it into the skills of their trainees. This is why the world’s top academic institutions provide full access to a variety of electronic and print sources for their faculty and students; libraries digitize their print collections and expand links to electronic periodicals, particularly to open-access journals.

With the advent and wide use of digital technologies for science communication in the last two decades, the concept of access to scholarly resources has changed. Given the dire need for information immediately accessible after publication, most established publishers launched digital repositories or e-libraries for their individual and institutional subscribers (eg, SpringerLink, Elsevier’s ScienceDirect, Wiley Online Library). The US National Library of Medicine went further and designed PubMed Central as a free digital library of full-texts indexed in PubMed and PubMed Central. Institutional and individual repositories have been launched and rapidly grown to facilitate access to scientific information, networking within professional communities, and research collaboration. Perhaps the best example of such an initiative is ResearchGate, a networking platform for scientists, which encourages sharing raw scientific data and published articles to change the global science discourse. Finally, many brand new open-access journals have appeared on the internet, followed by a trend of transforming subscription journals to fully or partially open-access media. In fact, most journals in small professional communities and non-mainstream science countries turned the challenge of digitization and open access into an opportunity of presenting their best research papers to the global community and advancing science in their countries.

The *Croatian Medical Journal* is one of the best examples of a national open-access journal, which made full-texts of its articles freely available online, reached out to the international community, got archived in PubMed Central, and increased its citation rates to the level of the journal with the highest impact factor in Southeast Europe (1,2). All Iranian biomedical journals (n=163) chose the open-access model, funded by national academic institutions, and some got archived in PubMed Central, which can be the driver for the scientific quality and impact of the journals. The open-access move in Iran can eventually translate into credibility and indexability of the journals, most of which struggle to get indexed by MEDLINE and Web of Science (3). Likewise, one-third of 187 Korean open-access medical journals indexed in the KoreaMed database are now archived in PubMed Central owing to the improved quality of editing and editorial policies encouraging open access (4). Indian scientists have recently improved the public awareness of open access, editors managed to index 407 open-access journals in the Directory of Open Access Journals (DOAJ), and 25 local institutional repositories got listed in the Registry of Open Access Repositories (ROAR) (5). Finally, a recent study indicated that the move toward open access is favored and sponsored by universities and professional societies in countries of Latin America, Eastern Europe, and Asia (6).

The open-access policy has also found strong supporters in developed countries, primarily among the large commercial publishers, who opted for various business models to facilitate free access and to increase the impact of publicly funded research. As a consequence, the United States with its 1302 open-access journals currently tops the list of countries in the DOAJ database (7). In the UK, the government support for open-access publishing has led to the adoption of the national strategy in 2012, making it possible to allocate a sizeable proportion of research funds to open-access publishing and archiving papers in publicly accessible digital repositories (8,9). As a consequence, the Research Councils UK, a coordinator for research funding, highlighted the intention to mandate publishing in journals with “comprehensive open access.” Importantly, one of the major research funders in the UK, the Welcome Trust, has already set a successful example by allocating funds for open access from 2007. The move toward comprehensive and immediate open access will take at least five years. In the meantime, the influential British media, and particularly *The Lancet* journals, have recently endorsed the strategy and provided options for open access, operating alongside the traditional subscription model (10). The BMJ Group launched the *BMJ Open* journal in 2011, which got indexed in PubMed and Web of Science, archived around 1400 quality items in PubMed Central, and received its first journal impact factor (JIF) of 1.58 in 2013. The rapid growth of *BMJ Open* is not surprising as the *PLOS One*, a flagship open-access journal of science and medicine published by Public Library of Science (US), has already reached a record of more than 69 000 articles, archived in PubMed Central since its launching in 2006, with the latest JIF of 3.73 and total cites of 133 246. These successful examples highlight the viability and impact of the new publishing model. The latter is also reflected in the constantly growing number of organizations mandating open access, with 177 institutions and 81 funding agencies being registered at the ROAR as of August 1, 2013 (11).

Open access is relatively new to the publishing market, and it is still shaping, with several of its options being experimented globally. In 2002, the Budapest Open Access Initiative issued a statement containing the first definition of open access (12). Its main point relates to unrestricted for any users online access to peer-reviewed journals, books, theses, presentations, and other forms of scholarly information. This definition also implies free distribution, copying, indexing, and various forms of lawful reuse without violation of the copyrights. To fully comply with the open access concept, journals and other forms of media have to secure funds alternative to the subscription and pay-per-view fees, improve technical quality and online readability of their output, and arrange its permanent archiving in a domain such as PubMed Central.

Research funding and article-processing charges (APC) turned to be the most difficult issues for most of the traditional or new journal publishers operating the open access model. The UK research funders, for example, mandated allocation of a fraction of their funds to open access, though this measure did not lead to an increase of total expenses for research projects. Such an initiative can influence the authors’ preferences of their target journals and boost the so-called gold open access, which requires payment for publishing and opening access immediately after the publication. Public Library of Science, BioMed Central, Dove Medical Press, and Libertas Academica are among the open-access publishers who successfully implemented the gold model through diverse sources of payments and avoided jeopardizing the quality of accepted papers. For example, BioMed Central has a reasonable article-processing charge of Ł1290, which can be discounted and waived for authors from low-income countries and for those lacking research funds. However, many other publishers, particularly those exploiting the “author-pays” option, have been strongly criticized for lowering publishing standards in an attempt to increase the number of papers with processed payments (13,14). One of the main arguments against the publishers corrupting the whole system of open access is that the lack of basic quality control by skilled reviewers and editors opens the gates for papers unacceptable for most journals with a strong infrastructure and high threshold for publication.

What may serve as a more sustainable and unbiased model for publishing, disseminating, and preserving scholarly papers is the green open access. Its main advantage is that the author publishes a paper in a journal and then self-archives its pre- or post-print version in an individual, institutional, specialist, or central repository such as PubMed Central. The green model is sustainable trough the subscription fees and other lawful financial sources. Its openness, however, varies, depending on the embargo period imposed on public archiving the contents after publication, which ranges from 6 to 24 months. Green open-access papers are usually published under a Creative Commons (CC) license, facilitating free distribution, reuse, and remix of the contents for non-commercial purposes with appropriate credits to the primary source (15). One of the inherent disadvantages of the green model is that the policy of self-archiving in various repositories substantially decreases online traffic of the primary sources of publications by directing the readers to the repositories, and particularly by increasing respective downloads from the PubMed Central platform (16). Simultaneous archiving in multiple digital repositories may also sophisticate the citation tracking in databases and search engines such as Google Scholar.

A large number of journals published by professional societies, which serve interests of small communities, generate essential revenue from advertisements and subscriptions. It seems that the hybrid open access is the most suitable publishing model for this set of journals, which diversifies financing through paid open access to separate articles and traditional income from subscriptions (17). The hybrid gold open access model is also widely implemented by large publishers of traditional “closed” journals such as Oxford University Press, Springer, Wiley, Elsevier, and Bentham Science Publishers. This move makes the journals preferable targets for researchers, who are obliged to open access to their publicly funded research, and maintains a pool of authors, who do not have funds for open access and rely on the traditional publishing model. Though the hybrid model seems advantageous and fair for publishers and authors, its costs and implementation may vary. It may also lead to unethical prioritization of paid open-access papers as well as rejection or delays with publication of non-paid papers (18).

Apparently, open-access publishing is becoming a global trend. The absolute majority of government agencies, universities, and professional societies are now transforming their subscription journals to open-access media, while most commercial publishers are launching a large number of brand new open-access journals (6). Such a move makes it important to evaluate the implications and to propose a publishing model adjusted to a specific scientific and business environment. Open-access publishing has brought about the concept of open peer review, which is widely operated by some of the well-established open-access publishers (eg, BioMed Central). If properly implemented, open peer review, as an option for quality control, may be also helpful for the transformation and international recognition of most journals currently blamed for predatory practice.

The implementation of open access has also changed webometric and bibliometric figures for most scholarly journals by attracting attention of the users to the easily accessible online contents. A controlled trial comparing full-text downloads and new visitors at the eleven journals published by the American Physiological Society found significant advantages of the articles made open in the first six month after publication over their subscription matches (19). When citations to these two sets of articles were analyzed within the first year after publication, no difference was found. Likewise, a large multidisciplinary study comparing 610 open-access journals with 7609 subscription journals in Web of Science and 1327 open-access journals with 11 124 subscription journals in Scopus found no difference in citation rates after controlling for discipline, age of the journal, and location of the publisher (20). This study, however, distinguished open-access journals with article-processing charges as more advantageous citation-wise, and particularly in terms of the two-year JIF than the same journals free to publish in. Thus, implications of open access are not merely linked to the ease of access and high retrievability of full-texts (21). Multiple factors influence the whole system, with the quality, relevance, and prestige of the published sources still remaining the major players in the field.

In conclusion, the open-access movement is gradually changing the ways of scientific research, literature search, journal editing, publishing, and archiving. Advances in digital publishing are forming the base for improved formatting, readability, and rapid distribution of research data. In the wake of changing the system of publishing, however, what concerns most is the quality of publications and unjustified rise of article-processing fees (22). While traditional attributes of science publishing such as unbiased peer review, selective journal indexing, and comprehensive ranking may be instrumental for guaranteeing credibility of the open-access models worldwide, secure research funding and more transparent financing of the open-access media may add to sustainability of future science publishing.
